# Investigating heterogeneous protein annotations toward cross-corpora utilization

**DOI:** 10.1186/1471-2105-10-403

**Published:** 2009-12-09

**Authors:** Yue Wang, Jin-Dong Kim, Rune Sætre, Sampo Pyysalo, Jun'ichi Tsujii

**Affiliations:** 1 Department of Computer Science, School of Information Science and Technology, University of Tokyo, Tokyo, Japan; 2 School of Computer Science, University of Manchester, Manchester, UK; 3 National Center for Text Mining, University of Manchester, Manchester, UK

## Abstract

**Background:**

The number of corpora, collections of structured texts, has been increasing, as a result of the growing interest in the application of natural language processing methods to biological texts. Many named entity recognition (NER) systems have been developed based on these corpora. However, in the biomedical community, there is yet no general consensus regarding named entity annotation; thus, the resources are largely incompatible, and it is difficult to compare the performance of systems developed on resources that were divergently annotated. On the other hand, from a practical application perspective, it is desirable to utilize as many existing annotated resources as possible, because annotation is costly. Thus, it becomes a task of interest to integrate the heterogeneous annotations in these resources.

**Results:**

We explore the potential sources of incompatibility among gene and protein annotations that were made for three common corpora: GENIA, GENETAG and AIMed. To show the inconsistency in the corpora annotations, we first tackle the incompatibility problem caused by corpus integration, and we quantitatively measure the effect of this incompatibility on protein mention recognition. We find that the F-score performance declines tremendously when training with integrated data, instead of training with pure data; in some cases, the performance drops nearly 12%. This degradation may be caused by the newly added heterogeneous annotations, and cannot be fixed without an understanding of the heterogeneities that exist among the corpora. Motivated by the result of this preliminary experiment, we further qualitatively analyze a number of possible sources for these differences, and investigate the factors that would explain the inconsistencies, by performing a series of well-designed experiments. Our analyses indicate that incompatibilities in the gene/protein annotations exist mainly in the following four areas: the boundary annotation conventions, the scope of the entities of interest, the distribution of annotated entities, and the ratio of overlap between annotated entities. We further suggest that almost all of the incompatibilities can be prevented by properly considering the four aspects aforementioned.

**Conclusion:**

Our analysis covers the key similarities and dissimilarities that exist among the diverse gene/protein corpora. This paper serves to improve our understanding of the differences in the three studied corpora, which can then lead to a better understanding of the performance of protein recognizers that are based on the corpora.

## Background

Named entity recognition plays an important first role in information extraction and text mining. Recently, due to the increase in biomedical research literature, biomedical NER (bio-NER), which aims at the automatic identification of gene and protein names, has become a research focus. Human-annotated corpora are widely used in the development of bio-NER systems. There are several well-known corpora that have gene/protein mention annotations, such as GENIA [1], GENETAG [2], AIMed [3], PennBioIE [4], etc. Based on these corpora, many protein mention recognizers have been developed, some of which are able to achieve state-of-the-art performance [5-7].

Nevertheless, a well-known problem remains. Since the protein annotations are made by different groups, and lack an explicit, unanimous rule that defines what should be annotated, it is likely that the annotations in the different corpora are incompatible.

The incompatibility among the corpora brings about several significant problems. For example, it is difficult to effectively utilize more than one corpus to develop a protein mention recognizer. There are very few high quality protein recognizers that are developed by utilizing multiple corpora, because it is unlikely for the recognizer to directly benefit from corpus integration. It is also difficult to compare systems that are developed using different corpora. Although there are many systems that recognize protein mentions from the PubMed text, the reported performance varies significantly, even for instance where the systems were developed with the same method. For example, methods based on conditional random fields (CRFs) report F-scores of 79.82% on GENIA [8] and 86.83% on GENETAG [9]. If each of the two recognizers were evaluated on the other corpus, the performance would be much lower [10]. Ultimately, the problems are largely caused by the incompatibility of different protein annotations, and cannot be resolved effectively without understanding the differences in the annotations.

In this paper, we explore the sources of incompatibilities among three well-known corpora with gene/protein annotations, GENIA, GENETAG and AIMed, and seek solutions to overcome the incompatibilities. We first observe the reduction in performance that results from using two of the three corpora together (as a single resource). Then, we carefully study the documentation of the three corpora, in order to determine the sources of incompatibility. Through a series of experiments, we quantify the incompatibility problems, while finding reasonable strategies to avoid the problems that are caused by the incompatibility of protein annotations. This study aims at analyzing the sources of incompatibility, and reducing the corpus inconsistencies.

## Results and discussion

Our research is based on the three selected corpora: GENIA, GENETAG and AIMed. Here, we present the findings from studying the documentation of the corpora, and from the results of our experiments. Refer to the Methods section for further details regarding preliminary experiments, significance tests, related works, and information on the corpora and on the protein mention recognizer used in this research.

### Characteristic of the corpora

We investigate three corpora that are often used in biomedical natural language processing (bio-NLP). Table 1 summarizes the differences between the three corpora, which are determined by analyzing the corpora's published literature.

**Table 1:** Characteristic of corpora

		AIMed	GENETAG	GENIA
Size	*abstracts*	225		1,999
	*sentences*	1,987	10,000	18,554

Entity	*scope*	human P/G	P/G/R	human P/G/R
	*number*	4,075	11,739	34,264(P)/10,002(G)/944(R)
	*coverage*	specific occurrence	specific occurrence	all occurrences
	*type*	no	no	Ontology
				7 types(P)/5 types(G)/5 types(R)

Legend:

Size: Number of abstracts or sentences in the corpus used in this research

Entity scope: Types of the named entities identified in the corpus: (P)rotein, (G)ene, (R)NA

Entity number: Number of the annotated in-scope entities in the corpus

Entity coverage: Coverage of in-scope entity occurrences in each sentence

Entity type: Explicit identification of the types of the annotated in-scope entities

All three corpora contain annotations that identify entities in the text, including proteins and genes; however, only GENIA contains information that specifies the types of the entities. This study benefits from the fine-grained protein annotation of GENIA. We compare the other two corpora to GENIA. GENIA aims at including an exhaustive annotation of entities of types that are relevant to the corpus, while the other two corpora have based entity annotation on a specific constraint that the tagged entity must ultimately be traceable to a specific gene/protein (e.g. "tumor necrosis factor 1" would be annotated, while "tumor necrosis factor" would not be annotated).

In terms of text organization, compared to GENETAG, GENIA and AIMed are closer to each other. GENIA and AIMed are focused on the "human" domain, but GENETAG covers a more general domain of PubMed. GENIA and AIMed exhaustively collect sentences in abstracts, but GENETAG collects sentences that are relevant to NER, in other words, GENETAG contains both true and false gene/protein names in a variety of contexts. In terms of text selection for entity annotation, GENIA and GENETAG are closer to each other, compared to AIMed. The former two corpora tend to select longer text fragments as entity references (see section on "Incompatibility one: boundary of protein mentions").

### Incompatibility of heterogeneous annotations

These differences suggest that combining the corpora into one training data will harm the performance of protein mention recognizers. We eventually identified four main sources of incompatibilities, which thoroughly explained the performance degradation when training with the united data. We applied corresponding strategies toward each aspect of the sources, to reduce performance degradation. Table 2 sums up the improvements of minimizing each of these negative effects.

**Table 2:** Improvement of minimizing the negative effects caused by the differences

Difference	Strategy	A	A+GENIA	G	G+GENIA
-	-	77.68	66.16	69.65	63.62

boundary of annotated entities	to loosen matching criterion	85.20	80.23	84.11	77.33

annotated entities of interest	to find compatible annotations		85.21		82.17

sentence selection	to select compatible sentences only		85.96		83.75

The scores shown in this table are the F-scores of training with the following data: A(IMed), A(IMed)+GENIA, G(ENETAG) and G(ENETAG)+GENIA.

As expected, the F-score performance, when training with combined data, was significantly lower than when training with the pure corpora. Based on the exact matching criterion (correct beginning and ending positions of the required annotations), the performance is degraded about 11.5% and 6.0%, when AIMed and GENETAG are respectively combined with the GENIA protein annotations as training data (the pure AIMed and GENETAG corpora are correspondingly used as test data, shown in the first data row of Table 2). (See the Methods section for details about these preliminary experiments, and for the experimental settings.)

The succeeding sections detail the sources of the incompatibility, and describe each of the improvements listed in Table 2. Ways to avoid the negative sides of the heterogeneities are also explained in detail.

### Incompatibility one: boundary of protein mentions

The selection of text spans, e.g. the beginning and ending boundaries, to be annotated is identified as one of the major sources for the incompatibility that exists for protein annotation across the three corpora.

Since it is difficult to determine whether the category of an entity, which occurs before or after the entity, can be considered as part of the entity name or not, the selection of boundaries is complicated [11]. For example, the English word "protein" can be treated differently in the following two text expressions, "p21ras protein" and "tumor suppressor protein". In the first case, "p21ras" is considered to be sufficient in naming the object. The term denoting the semantic category, "protein" is redundant, and may not be annotated as a part of the entity name. In fact, the decision to annotate "protein" in such a case often does not affect the utility of a NER system, because the system has correctly identified "p21ras" as a protein, and this information is adequate for mining the relationship between "p21ras" and another protein. Similarly, "the p21ras protein" or "the p21ras" could also be considered to be acceptable. However, in the second case, without "protein", "tumor suppressor" is inadequate in denoting the protein. The actual meaning is changed when omitting "protein". The semantic category "protein", which serves as an important clue, is quite essential in distinguishing this specific entity from a more general one. Thus, the inclusion of the word "protein" in the protein annotation is dependent on annotation scheme. GENIA and GENETAG almost always include the word "protein" in protein annotations, which indicates their schemes on words like "protein", but AIMed excludes "protein" in most cases.

The difference in boundary word selection between different protein annotation schemes can be measured in two ways: by calculating the average length of protein mentions and annotation entropy of boundary words.

#### Average length of protein mentions

The ambiguous annotation of boundary words is also a factor that affects the average length of the protein mentions in the three corpora. The average length per protein mention is 1.3 tokens in the AIMed corpus, 1.9 tokens in the GENIA corpus, and 2.1 tokens in the GENETAG corpus.

Many long protein mentions are introduced when we add the GENIA annotations into AIMed, creating another possible source of performance degradation in recognizing the shorter protein mentions in the AIMed corpus.

This observation suggests that the GENIA and GENETAG corpora are inclined to select more descriptive expressions for the protein annotation, in comparison with AIMed.

#### Annotation entropy of boundary words

In a given corpus, some words are annotated inside of protein mentions, while other words are not. The annotation entropy of boundary words is calculated by Formula (1).(1)

where *E*_*b *_denotes the annotation entropy of a given word, *P*_*a *_denotes the percentage of the occurrences of this word that is annotated, and  denotes the percentage of the occurrences of this word that is not annotated.

For the sake of brevity, the (boundary) "word" discussed in this section describes the word that appears at the beginning or end of an annotated entity, or describes the word that abuts an annotated entity. The value of the annotation entropy of a boundary word *E*_*b *_ranges from 0 (consistent) to 1 (inconsistent). When the annotation entropy of a boundary word *E*_*b *_is 0, this word is perfectly annotated (the word is always inside or outside protein names), and keeps this annotation consistency throughout the entirety of the corpus. On the contrary, when the annotation entropy of a boundary word *E*_*b *_is 1, the word's annotation is so disorderly (half of the occurrences of the word are inside and the remaining half of the occurrences of the word are outside protein names), that we can hardly find any rules about whether to regard this word as a part of the protein mentions or not.

In general, there are two types of ambiguous boundary words: descriptive adjectives, which usually occur before protein names as modifiers, such as "normal", "activated" or "human", and nouns, which usually occur after protein names as heads (such as "protein" or "molecule"). Some boundary words appearing in each corpus are listed in Table 3. In order to characterize the differences among the three corpora in terms of the annotation entropy of boundary words, the words with an annotation entropy close to 1 (in any one of the three corpora) were included in Table 3. The GENIA tagger [12] was used to determine the Parts-Of-Speech of the words. For the entire boundary words list, see "Additional file 1: the boundary words of the GENIA, GENETAG and AIMed corpora".

**Table 3:** List of boundary words

Category	Word	AIMed			GENIA			GENETAG		
		***N***_***a***_	***N***_***n***_	***E***_***b***_	***N***_***a***_	***N***_***n***_	***E***_***b***_	***N***_***a***_	***N***_***n***_	***E***_***b***_
Adjective	constitutive	0	0	0.0000	12	11	0.9986	2	2	1.0000
	endogenous	0	0	0.0000	22	11	0.9183	9	9	1.0000
	exogenous	0	0	0.0000	9	16	0.9427	2	3	0.9710
	inducible	0	0	0.0000	18	17	0.9994	0	0	0.0000
	low	0	0	0.0000	14	11	0.9896	2	3	0.9710
	major	0	0	0.0000	25	15	0.9544	1	5	0.6500
	putative	0	0	0.0000	15	15	1.0000	0	0	0.0000
	recombinant	1	8	0.5033	36	24	0.9710	26	2	0.3712
	soluble	1	10	0.4395	14	15	0.9991	1	4	0.7219
Noun before	factor	0	0	0.0000	5	26	0.6374	17	15	0.9972
	plasma	0	0	0.0000	13	1	0.3712	17	12	0.9784
	protein	12	34	0.8281	159	18	0.4743	53	10	0.6313
Noun after	form	0	0	0.0000	21	14	0.9710	0	0	0.0000
	pathway	0	0	0.0000	0	0	0.0000	8	10	0.9911
	protein	40	17	0.8791	794	14	0.1262	241	2	0.0688

Here, "Noun before" indicates the noun occurring before an entity as a modifier, "Noun after" indicates the noun occurring after an entity as a head. *N*_*a *_represents the number of the annotated occurrences, and *N*_*n *_represents the number of un-annotated occurrences.

From Table 3, the boundary annotation problem appears for various words. The distribution of these words is diverse, particularly for adjectives. Since the number of characters tagged in the AIMed corpus was kept to a minimum, only the names of protein mentions were annotated, most of which are proper nouns, and most of the adjectives were not annotated. However, the developers of the GENIA corpus followed an annotation strategy for which generic terms were annotated. In the GENIA annotation scheme, specifiers (determiners, ordinals or cardinals) do not appear in tagged entities, but qualifiers, including adjectives and noun modifiers, remain. The adjectives before the protein mentions are annotated only if they are required for clarifying the meaning of the protein mentions (e.g. in the protein mention of "inducible cAMP early repressor", "inducible" is annotated, because it is needed to understand the meaning of the protein mention). Furthermore, the GENETAG annotators chose some semantic constraints, which state that the tagged entity must contain its true meaning in the sentence context (e.g. the word "receptor" is necessary in differentiating "IGG receptor" from "IGG", which is an important semantic distinction). These constraints were geared towards multi-word entities, and especially for entities that include numbers, letters and acronyms.

Because the conventions on boundary words vary significantly, to prevent underestimating performance of protein mention recognizers, we need an alternative matching criterion, other than the exact matching. To provide alternative evaluation perspectives, researchers have developed a variety of evaluation criteria that loosens the matching to varying degrees. Partial matching is also considered [13,14] (if any part of a protein mention is identified, it will be considered to be a correct answer), because it has previously been shown to capture the presence of an entity with disregards to its exact textual representation. According to this partial matching criterion, the performance for the pure and combined data are shown in the second data row of Table 2. Though by loosening the matching criterion, the performance of the training with the combined data still can not compete with the performance of the training with the pure data, under-estimation can be avoided.

### Incompatibility two: scope of the entities of interest

Although all three corpora include gene/protein mention annotations, the target tasks are different. The GENIA corpus aims at providing linguistically rich annotations on biological expressions. The GENETAG annotation follows a wide definition with the constraint that a gene/protein entity annotation must refer to a specific entity. The AIMed annotation focuses on extracting interactions among individual proteins. This difference has affected the scope of the annotated proteins: *GENIA is concerned with all of the protein-mentioning terms, GENETAG is based on specific genes/proteins, while AIMed focuses only on references from individual proteins*.

#### Categories of annotated entities

The extent to which the proteins annotated in the GENIA corpus is defined in the GENIA ontology [15]. In addition to the protein class, other classes such as *DNA*, *RNA*, *cell_line *and *cell_type *are also included. Further, the protein class is categorized into seven subclasses: *Protein_complex*, *Protein_domain_or_region, Protein_family_or_group*, *Protein_molecule*, *Protein_substructure*, *Protein_subunit *and *Protein_ETC*. Thus, in GENIA, "protein" is defined to include these seven concepts.

For AIMed, the scope of the proteins annotated is described by the following statement in the tagging guidelines: generic protein families are not tagged, only specific names that could ultimately be traced back to specific genes in the human genome are tagged [16]. That is, for example, "tumor necrosis factor" would not be tagged, while "tumor necrosis factor alpha" would be tagged.

Finally, based on the gene names from GenBank [17], the GENETAG annotations include domains, complexes, subunits, and promoters when the annotated entities refer to specific genes/proteins.

Hence, for the scope of annotated proteins, the documentation of the three corpora explicitly states that:

(1) The mentions of protein families are annotated in GENIA, but not in AIMed.

(2) Individual proteins (*Protein_molecule*) are annotated in all of the corpora.

(3) Both GENIA and GENETAG contain protein domain, complex and subunit annotations.

#### Compatible protein annotations

The published literature of the three corpora provides three aspects of the inclusion/exclusion of annotations of some classes of the GENIA protein subcategories. For example, for AIMed and GENIA, Item (1) and Item (2) relate to the *Protein_molecule *and *Protein_family_or_group *annotations. For GENETAG and GENIA, Item (2) and Item (3) relate to the *Protein_molecule*, *Protein_domain_or_region*, *Protein_complex *and *Protein_subunit *annotations. However, there are other protein subcategories annotated in GENIA; and so far, we are unable to find any clue regarding the inclusion or exclusion of these protein subcategories in the scope of the annotations in AIMed or GENETAG. For instance, it is unclear whether the GENETAG annotations are compatible with the GENIA *Protein_family_or_group *annotations or not. It is also unclear whether the GENIA *Protein_domain_or_region*, *Protein_complex *and *Protein_subunit *annotations are compatible with the AIMed annotations. We performed a series of experiments to confirm the three aspects that we found, and to find other clues on the other protein subclasses.

For training, we used each of the GENIA protein subclasses, one by one, together with the AIMed corpus and the GENETAG corpus (in separate experiments for each). Each time, we regarded the annotations from a different GENIA protein subclass as positive examples. The experimental results are listed in Table 4.

**Table 4:** Experimental results of the GENIA protein subcategory annotations plus AIMed and GENETAG, respectively

		AIMed + GENIA			GENETAG + GENIA		
Matching criterion	Protein subcategory	Precision	Recall	F-score	Precision	Recall	F-score
Exact	complex	76.60	41.38	53.73	77.27	27.97	41.07
	domain_or_region	78.74	41.63	54.47	75.94	27.46	40.34
	ETC	81.38	40.74	54.30	78.89	26.58	39.77
	family_or_group	68.49	41.63	51.79	73.58	34.61	47.08
	molecule	81.83	66.16	73.16	74.78	47.99	58.46
	substructure	81.27	41.00	54.50	78.46	26.52	39.64
	subunit	82.40	43.04	56.54	79.16	27.53	40.85

Partial	complex	91.96	49.68	64.51	92.89	33.63	49.38
	domain_or_region	88.41	46.74	61.15	90.45	32.71	48.05
	ETC	91.58	45.85	61.11	93.77	31.59	47.26
	family_or_group	81.09	49.30	61.32	88.62	41.69	56.70
	molecule	91.47	73.95	81.78	92.40	59.30	72.24
	substructure	91.39	46.10	61.29	93.99	31.76	47.48
	subunit	91.93	48.02	63.09	93.77	32.61	48.39

As indicated on the table, it was found to be the most harmful to combine the GENIA *Protein_family_or_group *annotations together with AIMed, which supports Item (1): the mentions of protein families are annotated in GENIA, but not in AIMed. Also, Item (2) is confirmed by the results that the GENIA *Protein_molecule *annotations least negatively affect the performance in recognizing the proteins tagged in the AIMed and GENETAG corpora. It also indicates that the GENIA *Protein_molecule *definition is the closest to the GENETAG and AIMed protein definitions, when compared with other GENIA protein subcategories.

Furthermore, adding the GENIA *Protein_subunit*, *Protein_complex *and *Protein_domain_or_region *annotations also helps to recognize the AIMed and GENETAG annotated proteins (mentioned in Item (3)). Because there are only a few *Protein_substructure *and *Protein_ETC *annotations in GENIA (103 and 85, respectively), the two protein subcategories are excluded from further consideration. In addition to the three mentioned aspects, we also found that besides the *Protein_molecule *annotations, the *Protein_family_or_group *annotations were the most helpful for our recognizer to find the GENETAG protein annotations.

We further observe that when adding only the GENIA protein subcategory annotations to a pure corpus, the precision on both AIMed and GENETAG is quite high, but the recall is very low. This observation suggests that if we also add the other helpful protein subclass annotations into the training material, we might improve the recall, while maintaining a reasonable level of precision. The No.1 and No.2 data blocks in Table 5 show the experimental results based on this hypothesis. The experimental results show that when we collectively use the helpful GENIA protein subclass annotations, the recall improves significantly, while minimizing the decrease in precision. For ease of comparison, the preliminary experimental results mentioned in the Methods section are also listed in the last two data blocks of Table 5.

**Table 5:** Experimental results of the GENIA subcategory annotations plus AIMed and GENETAG, respectively

**No**.	Data	Criterion	Precision	Recall	F-score
1	AIMed+ molecule+ subunit	Exact	81.69	66.67	73.42
		Partial	91.39	74.58	82.14
	AIMed+ molecule+ subunit+ complex	Exact	77.78	66.16	71.50
		Partial	91.14	77.52	83.78
	AIMed+ molecule+ subunit+ complex+ domain	Exact	76.45	67.18	71.52
		Partial	89.39	78.54	83.62

2	GENETAG+ molecule+ family	Exact	71.08	55.03	62.03
		Partial	87.40	67.66	76.27
	GENETAG+ molecule+ family+ subunit+ complex+ domain	Exact	69.90	58.28	63.56
		Partial	85.30	71.11	77.56

3	AIMed+ DNA	Exact	75.69	41.76	53.83
		Partial	87.96	48.53	62.55
	AIMed+ filtered DNA	Exact	80.48	42.66	55.76
		Partial	92.29	48.91	63.94

4	GENETAG+ DNA	Exact	69.82	36.44	47.89
		Partial	83.97	43.82	57.59
	GENETAG+ filtered DNA	Exact	76.57	35.18	48.21
		Partial	92.70	42.60	58.38

5	AIMed+ molecule+ subunit+ complex+ filtered DNA	Exact	77.49	67.69	72.26
		Partial	91.37	79.82	85.21
	AIMed+ molecule+ subunit+ complex+ domain+ filtered DNA	Exact	75.78	68.33	71.86
		Partial	89.09	80.33	84.49
	GENETAG+ molecule+ family+ subunit+ complex+ domain+ filtered DNA	Exact	70.97	66.14	68.47
		Partial	85.14	79.34	82.14
	GENETAG+ molecule+ family+ subunit+ complex+ domain+ filtered DNA+ RNA	Exact	71.34	67.02	69.11
		Partial	84.82	79.68	82.17

6	pure AIMed	Exact	80.44	75.10	77.68
		Partial	88.24	82.38	85.20
	AIMed + GENIA Protein	Exact	65.06	67.31	66.16
		Partial	78.89	81.61	80.23

7	pure GENETAG	Exact	71.38	68.00	69.65
		Partial	86.21	82.12	84.11
	GENETAG + GENIA Protein	Exact	69.75	58.48	63.62
		Partial	84.77	71.08	77.33

Here, DNA represents the GENIA *DNA_domain_or_region *and RNA represents the GENIA *RNA_molecule*.

Since our goal is to find a way to prevent the system performance degradation, we set the performance of the pure AIMed or GENETAG corpus training as the (minimum) goal. Then, the potential (maximum) reduction rate of incompatibility can be calculated by using Formula (2):(2)

where *R*_*e *_denotes the corpus incompatibility reduction rate of a given experiment, *F*_*e *_denotes the F-score of the given experiment, *F*_*r *_denotes the reference F-score of the pure source corpus training (e.g. AIMed or GENETAG only), and *F*_*b *_denotes the baseline F-score of the training with the pure source corpus plus the GENIA protein annotations.

By combing the AIMed corpus with the GENIA *Protein_molecule*, *Protein_subunit *and *Protein_complex *annotations, we reduced the corpus incompatibility by 71.43%, based on the partial matching criterion. (We adopt the looser criterion for calculating the reduction rate of incompatibilities, but also provide the exact matching scores, for reference.) Thus, when combining the annotations from the GENIA corpus with the AIMed corpus, we can use the annotations of these three protein subclasses, since they were found to be compatible to some extent.

Moreover, if we combine the GENETAG corpus with the GENIA *Protein_molecule*, *Protein_subunit*, *Protein_domain_or_region*, *Protein_family_or_group *and *Protein_complex *annotations, the corpus incompatibility reduction rate (3.39%) is not as notable as the rate achieved on AIMed (71.43%). Subsequently, other than the five protein subcategories, there might be other category annotations that are compatible with the GENETAG annotations. This situation will be explored in the following paragraphs.

We found sentences that include GENIA *Protein_subunit*, *Protein_complex *and *Protein_domain_or_region *annotations, which will not cause an incompatibility during corpus combination. That is, in GENIA, these entities are regarded as proteins that are compatible with the AIMed and GENETAG annotations, so we can introduce most of the GENIA annotations of these entities into AIMed or GENETAG without causing a negative influence. Some examples are shown (see Appendix). In order to show the original corpus annotation, all of the entity annotation types are presented.

#### Ambiguity between DNA and genes

The annotations in the AIMed and GENETAG corpora also include some gene names, without differentiating them from proteins. For the GENIA corpus, the *Protein *annotation is applied only to proteins, while genes are annotated in the scope of *DNA *annotations. Treating gene annotations in the GENIA corpus in the same way as for the AIMed and GENETAG corpora would improve the consistency; nevertheless, the GENIA annotation does not include explicit gene annotation. Instead, genes are annotated as instances of *DNA_domain_or_region*, which is also applied to other DNA regions (e.g. binding sites and c-terminals). We assume that if the *DNA_domain_or_region *annotations that are not pure genes can be filtered out from *DNA_domain_or_region *annotations, examples in the remaining GENIA *DNA_domain_or_region *annotations will positively affect the corpus combination. Consequently, if we assume that the performance of a recognizer trained with the AIMed corpus is sufficiently good, it will find most of the gene mentions in the GENIA corpus, based on the gene definitions in AIMed. The other *DNA_domain_or_region *annotations that are not compatible with the AIMed gene definitions will then be filtered out. (This filtering would only work perfectly if the performance of the recognizer was perfect, so it will be a rough filtering.) The true positives, which are annotated as *DNA_domain_or_region *in the GENIA corpus and also detected by the recognizer, will include *DNA_domain_or_region *instances, which are "AIMed-like" genes. In a similar way, we can also find the "GENETAG-like" gene mentions.

To examine the performance of the filtering, we added all of the *DNA_domain_or_region *annotations into the training set in one experiment, and only added the filtered "genes" into the training set of another experiment. The results (as indicated in the No.3 and No.4 data blocks in Table 5) show the disambiguation between DNA and genes works, although the improvement resulting from the filtering is modest, the improvement was relatively small for GENETAG. This may be due to the fact that, in addition to genes and proteins, the GENETAG corpus also includes RNA, domains, complexes, sequences, fusion proteins, etc. No distinction is made between these classes, so the filtering on GENETAG is more difficult than filtering on AIMed. The simple filtering cannot effectively filter out most of the real genes from so many other classes; however, simple filtering helps to filter out some *DNA_domain_or_region *annotations that are not necessary for the tagger. In fact, even some well-known machine learning algorithms did not perform well against a human-labeled model, even though human experts could not achieve a high agreement rate on protein, gene and RNA labels [18].

As mentioned earlier, adding only the *Protein_molecule*, *Protein_subunit *and *Protein_complex *annotations from GENIA resulted in the best performance for the AIMed corpus thus far. Then, in addition to the three annotation types, we also added the filtered *DNA_domain_or_region *annotations to train our protein mention recognizer. The same experiment was performed with GENETAG. The experimental results are shown in the first three rows of the No.5 data block in Table 5. In comparison the first two rows of the No.5 data block with the No.6 data block in Table 5, we can tell that the corpus incompatibility between GENIA and AIMed is removed completely, because our current best result on AIMed (85.21) is better than the performance of training with the pure AIMed corpus (85.20). In Comparing the third row of the No.5 data block with the No.7 data block in Table 5, in the case of GENIA and GENETAG, the corpus incompatibility was reduced by 70.94%.

#### RNA annotations in GENETAG

In addition to the GENIA annotations that have already been confirmed to be compatible with the GENETAG annotations, there is another GENIA subcategory that should contain compatible annotations: namely the *RNA_molecule*.

In addition to the six subcategory annotations mentioned above, we have also added the GENIA *RNA_molecule *annotations to the GENETAG corpus. The experimental results are shown in the last row of the No.5 data block in Table 5. By comparing the F-scores shown in the last two rows of the No.5 data block in Table 5, we can see that the reduction of the incompatibility on GENETAG was improved by adding the *RNA_molecule *annotations.

The improvements thus far are shown in the third data row of Table 2. After we have explored as many compatible annotations between the corpora as possible, we can observe that the F-score performance gap between the pure source corpus training, and the GENIA-combined training is about 1.9% in F-score on GENETAG alone. For AIMed, the performance of the GENIA-combined training competes with that of the pure source corpus training.

### Incompatibility three: sentence selection

Although all of the possible compatible GENIA annotations have already been explored, there are still some sources that are responsible for the remaining incompatibilities between GENIA and GENETAG. When some sentences with the compatible annotations are introduced, some incompatible "missing" or "extra" annotations in these sentences may also be included.

To confirm this assumption, we applied a sentence selection policy. For a given subcategory, sentences that only contain annotations of this subcategory are selected for our corpus integration experiment. For instance, in the case of *Protein_molecule*, *DNA_domain_or_region *and *RNA_molecule*, with the exception of these three subcategories, if there are no other *Protein *subcategory, *DNA *subcategory, *RNA *subcategory or *Peptide *annotations in a sentence, then that sentence will be combined with AIMed or GENETAG as the training data. The results of the experiments with the selected sentences are shown in Table 6.

**Table 6:** Experimental results with the selected GENIA sentences plus AIMed and GENETAG, respectively

Experiment	Data	Size	Criterion	Precision	Recall	F-score
Exp 1	AIMed+ molecule+ subunit+ complex+ filtered DNA	7,433	Exact	76.67	70.50	73.45
			Partial	89.72	82.50	85.96
Exp 2	AIMed+ molecule+ subunit+ complex+ domain+ filtered DNA	7,771	Exact	77.03	71.52	74.17
			Partial	89.27	82.89	85.96

Exp 3	GENETAG+ molecule+ family+ subunit+ complex+ domain+ filtered DNA	7,771	Exact	71.73	66.85	69.20
			Partial	86.81	80.90	83.75
Exp 4	GENETAG+ molecule+ family+ subunit+ complex+ domain+ filtered DNA+ RNA	7,675	Exact	71.58	67.22	69.33
			Partial	86.30	81.04	83.58

The second column only shows the number of the selected GENIA sentences.

As evidenced in Table 6, adding 7,771 selected GENIA sentences with the *Protein_molecule*, *Protein_subunit*, *Protein_complex*, *Protein_domain_or_region *and the filtered *DNA_domain_or_region *annotations, with the partial matching criterion, further obtained a better performance with the corpus integration than the results obtained by training on the pure AIMed corpus. Moreover, we can see that combing the selected GENIA sentences with GENETAG also helped our protein mention recognizer work better on the GENETAG corpus. The corpus incompatibility reduction rate improves to 94.69%.

We also summarize the improvement obtained by the sentence selection strategy in the last row of Table 2. The incompatibility between AIMed and GENIA has then been eliminated completely, but for GENETAG and GENIA, a very small performance degradation remains (0.36% in F-score). The cause of the remaining incompatibility between GENETAG and GENIA will be explained in the next section.

Since sentence selection reduces the number of potentially incompatible examples that were added, the reduced incompatibility may be explained by the down sampling involved. To verify that the improvements are really achieved by sentence selection, we conducted experiments with randomly selected sentences. The same number of sentences were randomly selected from GENIA, as introduced in the sentence selection experiments, and are repeated 1,000 times. We then calculated the confidence intervals of the partial matching F-scores at the 95% confidence level. The statistical results are shown in Table 7. Since the F-scores of the sentence selection experiments fall outside of the range of the random sentence selection F-scores, sentence selection works on both the AIMed and GENETAG corpora.

**Table 7:** Statistical analysis of random sentence selection experiments at the 95% confidence level

Random experiment	Mean	Confidence interval	True mean range	F-score of sentence selection experiment
Exp 1	85.38	± 0.04	85.34 to 85.42	85.96
Exp 2	84.87	± 0.04	84.82 to 84.91	85.96
Exp 3	82.80	± 0.02	82.78 to 82.82	83.75
Exp 4	82.91	± 0.02	82.89 to 82.93	83.58

The experiments are as described in Table 6, with the randomly selected GENIA sentences.

### Incompatibility four: non-overlapping data

The results thus far indicate that incompatibility still exists among the corpora; in particular, the performance of training with GENETAG and GENIA is still a little lower than the performance achieved by using only GENETAG. Since the sentences in the three corpora are collected by different means, it is assumed that the proteins mentioned in the three corpora are mainly heterogeneous, which results in incompatibility.

To quantify this assumption, we examined the ratio of overlapping entities among different portions of each corpus and among different corpora. We first divided each corpus into two disjoint portions of the same size. In order to make the comparisons, we down-sampled sentences in each portion of GENIA and GENETAG, so that the gene/protein mentions in each portion are of the same number as the ones in one AIMed portion (2039). The numbers of overlapping entities in and across the corpora are shown in Table 8. Table 8 shows that the proteins in the GENETAG corpus are more heterogeneous than the proteins in GENIA and AIMed. In comparing the number of the intra-corpus overlapping entities with that of the inter-corpora overlapping entities, it further shows that the proteins across any two different corpora are significantly more heterogeneous than the proteins in a single corpus, which supports our assumption. We can therefore conclude that the heterogeneity of the proteins in the three corpora is another source of incompatibility. The incompatibility, however can be considered as a potential benefit of using all three corpora, rather than as a problem. The low overlapping ratio of the annotated entities in the three corpora implies that no single corpus can represent the annotation for the entire protein. Then, developing a NER system that can show a good performance on all three corpora would be ideal; this will remain a task for our future work.

**Table 8:** Overlapping entities intra-corpus and inter-corpora

	The number of overlapping entities			
Data	AIMed	GENETAG	GENIA-AIMed	GENIA-GENETAG
AIMed	449	113	117	-
GENETAG	113	204	-	108
GENIA-AIMed	117	-	347	-
GENIA-GENETAG	-	108	-	332

"GENIA-AIMed" and "GENIA-GENETAG" represent the GENIA data used in Exp 2 and Exp 4 of Table 6, which are compatible with AIMed and GENETAG, respectively. The number of gene/protein mentions in each portion is 2039.

## Conclusion

We have presented a comparative evaluation of protein annotations, by studying the GENIA, GENETAG and AIMed corpora. Our preliminary experiments showed a major performance degradation in protein name identification, when using the combined corpora as the training material (according to the exact matching criterion, the F-score performance dropped about 12% on AIMed and 6% on GENETAG, referring to section: "Preliminary experiments"). The documentation of these corpora was studied to identify the corpus heterogeneities that caused the performance degradation; in effect, a series of experiments that aimed at removing or avoiding the negative effects of the corpus differences were performed. We also strove towards grasping a better understanding of the different protein annotations implemented in the corpora.

Although all of the corpora are distributed with gene/protein annotations, the target entities are significantly different depending on the documentation of the corpora. The emphasis on individual proteins (*Protein_molecule*) involved in protein-protein interactions in the AIMed corpus is responsible for more than half of the disagreements between AIMed and GENIA. The scope of the proteins defined for the protein annotations to GENETAG and GENIA is the biggest source of the incompatibilities found between the two corpora: besides protein, the protein definition of GENETAG also includes gene, DNA and RNA, while the protein definition of GENIA does not. Even within a single corpus, the boundary word annotation is not guaranteed to be consistent (see Additional file 1). Finally, the low overlapping ratio of the annotated entities between each corpus is another reason for the incompatibilities.

These observations suggest the difficulty of benefiting from an increased size in training data by merging differently annotated corpora, unless the annotations of individual collections are adequately compatible. To address this issue, we proposed ways of avoiding the heterogeneities that we have already discovered.

We showed that the proposed methods removed the incompatibilities between AIMed and GENIA, thus showing an improved NER performance by combining the two corpora for training. The F-score performance improves from 80.23 to 85.96 based on the partial matching criterion, which makes the performance competitive with the performance of the pure AIMed corpus training (85.20). For GENETAG and GENIA, we also removed a significant amount of inconsistencies and achieved a comparable NER performance when combining the two corpora for training. The F-score performance improves from 77.33 to 83.75, merely 0.36% lower than using the pure GENETAG corpus training. This may be attributed in part to the fact that the GENETAG corpus is quite optimized for machine learning with a balanced number of positive and negative examples, while the GENIA corpus is not optimized. All improvements are proved to be statistically significant.

As the increasing amount of data helps to improve NER system performance, the creation of standard annotation guidelines proves to be an important task. The study done through this work provides insight to the existing protein annotations, which should be helpful for producing better annotation guidelines.

## Methods

### Preliminary experiments

We performed two preliminary experiments with AIMed and GENIA in order to confirm the following two assumptions: first, that we can improve the performance of a protein mention recognizer by increasing the size of the training data set; and second, that the system performance will drop when incompatible annotations are introduced into the training data set.

We divided the AIMed corpus into two parts: 80% for training and the remaining 20% for testing. In the first experiment, we only used the AIMed training portion. In this experiment, we performed eight sub-experiments, each time adding 10% more AIMed abstracts into the training portion. In the second experiment, in addition to the AIMed training portion, we also added all of the GENIA protein annotations. In order to keep the same increasing rate with GENIA as with AIMed, we also added the GENIA corpus in increasing proportions. In both experiments, we performed evaluation on the AIMed testing portion. In this paper, all evaluations on AIMed are carried out on the AIMed testing portion (20% of the corpus), unless otherwise noted. Also, for the sake of simplification, we refer to the AIMed training portion (80% of the corpus) as the "AIMed corpus".

**Figure 1:**
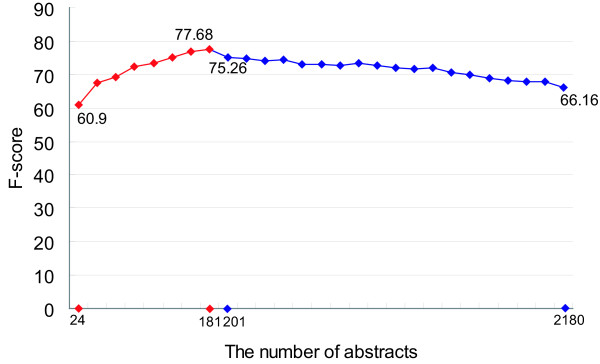
**Learning curve drawn from the results of the preliminary experiments with the AIMed and GENIA corpora**. The numbers represent the F-scores based on the exact matching criterion. The red color indicates the experimental results of the training with AIMed, and the blue color indicates the experimental results of adding GENIA. The F-score of 60.9% was obtained when about 10% of the AIMed abstracts were used for the training. Following that, 10% more AIMed abstracts were added to the training material gradually. Note that the best result (77.68% in F-score) was achieved when all the AIMed training portions (181 abstracts) were used. When 20 more GENIA sentences were added to the training material, the F-score degraded to 75.26%. The performance kept degrading when the GENIA abstracts were added in increasing proportions. Further, when the entire GENIA corpus (1,999 abstracts) was included, the F-score was as low as 66.16%.

A learning curve drawn from the results of the two experiments, mentioned above, is shown in Figure 1. The learning curve is still increasing, even after we used all eight training portions of the AIMed corpus. We would expect a further improvement if we were able to add more training data. When we actually added the protein annotations from the GENIA corpus to the training data set, a very small portion of GENIA was used (20 abstracts, one-ninth of AIMed); we witnessed performance degradation. When we added the entire GENIA corpus, which is more than ten times larger than the AIMed corpus, the performance degradation was found to be drastic. We assume that the degradation is caused by the incompatibility of the protein annotations in the two corpora, and we further assume that by decreasing the incompatibility, the learning curve will continue to increase, or will level out and remain constant.

We conducted two similar experiments with GENETAG and GENIA. For training, first, only the "GENETAG Train" subset (third-eighths of the corpus) was used, and then "GENETAG Train" subset plus the GENIA protein annotations were used. The evaluations were carried out on the "GENETAG Test" subset (one-eighth of the corpus). Unless otherwise specified, the "GENETAG ROUND1" subset (one quarter of the corpus) was not used. The other quarter of the corpus is not publicly available. In this paper, all evaluations on GENETAG are performed on the "GENETAG Test" subset only, and the "GENETAG Train" subset is simply called the "GENETAG corpus", except where explicitly noted. Again, the experimental results verified our assumption that the integration of the two corpora with heterogeneous annotations will lead to a performance degradation.

We additionally performed more experiments to further confirm our assumption. We split the GENIA corpus on the abstract level, so that 90% was used for training and the remaining 10% was used for testing. We trained our tagger with the GENIA training portion, and with the GENIA training portion plus AIMed and GENETAG, respectively; we then applied the tagger to the GENIA testing portion. The performance of the training with the integrated data was worse than the result from the training with the GENIA training portion, even if the added corpus is as small as AIMed (about 10% of GENIA). Such results further show that merging two heterogeneous corpora will degrade the performance, no matter how small one of the corpora is.

Further, we trained our tagger with two compatible corpora, by merging AIMed and GENETAG. AIMed and GENETAG were respectively evaluated by this trained tagger, in order to show that merging two corpora with homogeneous annotations does not guarantee that the corpus integration will avoid a degradation in the performance.

All results of the mentioned experiments are represented in Table 9.

**Table 9:** F-score performance of the inter- and intra corpus experiments according to the exact matching criterion

Testing data	Training data		
	AIMed	GENETAG	GENIA
AIMed	77.68	69.34	66.16
GENETAG	68.96	69.65	63.62
GENIA	78.36	77.80	78.76

"Testing data" represents the corpus used for evaluation. "Training data" represents the corpus which was "added" to the training portion of the "Testing data" corpus for training. For example, the score in the cell AIMed-GENETAG represents the performance of training on the training portion of the AIMed and GENETAG corpora, and testing on the testing portion of AIMed.

### Significance tests

For empirical natural language processing, on some test data set, researchers often evaluate whether some new technique improves the results, when compared to some current technique. When the new technique yields a better result, we must decide whether these result differences are due to the improvements in the new technique, or whether the improvements are based on chance. [19] offers some methods for statistically computing significant differences in the balanced F-score metric: by using computationally intensive randomization tests, and in particular, by using bootstrap over test set [20-22].

However, the bootstrap variances in elementary experiments were too high to conclude anything, so we instead used a McNemar paired test on labeling the disagreements [23-25]. It is over-optimistic to measure NER with the accuracy rate for individual labeling decisions, but for the statistical significance tests, individual labeling errors provide a more convenient basis.

Our protein recognition task labels each word with a label that indicates whether the word is the beginning of a protein (B), or the internal of a protein (I), or a general English word (O). With McNemar's test, we compare the correctness of the labeling decisions. A null hypothesis is that the disagreements (correct vs. incorrect) are due to chance. Table 10 summarizes the results of the tests between our best result and the pure corpus training result. These tests suggest that the system performance improves on AIMed and GENETAG, by the introduced techniques. The experiments are statistically significant.

**Table 10:** McNemar's tests on labeling disagreements

Null hypothesis	P-value
Exp1 vs. AIMed	2.04e-09
Exp2 vs. AIMed	3.57e-11

Exp3 vs. GENETAG	0.0254
Exp4 vs. GENETAG	0.00013

The experiments are as described in Table 6. AIMed and GENETAG represent the experiments with the pure AIMed and GENETAG corpora, respectively.

### Related works

Portability and reusability make machine learning attractive: given the same entity types and similar text types, for example, a protein name recognizer trained on one corpus would be able to recognize proteins in another corpus. However, incompatibility among corpora weakens the portability and reusability, as mentioned earlier.

To eliminate the negative influence of the inconsistencies, [26,27] converted several corpora into one unified format, without altering the semantics of the corpus. Furthermore, some methods have been proposed, aiming at improving the system performance on one corpus relatively different from the training corpus (e.g. domain adaptation [28], transfer learning [29], etc). None of the methods dealt with the essential distinctions between the training and testing corpora. [30] compared five corpora annotated for protein names, and analyzed the cause for the boundary errors and for the increased number of false positives. However, [30] did not explore the classification of proteins, and some of the five corpora are not so widespread today.

We previously studied the AIMed and GENIA corpora [31]. In this work, we extended our analysis to also cover the GENETAG corpus.

### Materials

Here, we briefly introduce the corpora selected in this research, and focus on their size and covered domains. Finally, we describe the protein mention recognizer used in our work.

#### The GENIA corpus

The GENIA corpus (version 3.02) [32] is a collection of articles extracted from the PubMed database with the MeSH terms: "human", "blood cells" and "transcription factors". There are 1,999 abstracts and 18,554 sentences in total. The corpus has been annotated with various levels of linguistic and semantic information. The term annotation is based on a taxonomy of 48 classes that are established on a chemical classification. Among the classes, 36 terminal classes were used to annotate the corpus. The total number of annotated terms/entities is 94,639. A simplified version called the JNLPBA corpus [33] is also used in the biomedical text mining domain. In recent years, the GENIA corpus has become one of the most frequently used corpora in the biomedical domain [34,35], and has been widely used for training natural language processing (NLP) tools such as NER [36] and relation miner [37].

#### The GENETAG corpus

The GENETAG corpus [38], which was used for the BioCreAtIvE Competition Task 1A [39], is described as the tagged gene/protein names in the PubMed text. The corpus was designed to contain sentences both with and without gene/protein names, in a variety of contexts. Gene/protein names are defined widely, but are subject to the specificity and the semantic constraints. The annotation guidelines were designed to allow flexible matching to the gold standard, while retaining the true meaning of the tagged entities.

There are 20,000 sentences and a total of 23,996 gene/protein names annotated in the GENETAG corpus. An additional file of 17,531 acceptable alternatives to the tagged gene/protein names is made available. The 20,000 sentences were split into four subsets called Train (7,500 sentences), Test (2,500 sentences), Round1 (5,000 sentences) and Round2 (5,000 sentences). With the exception of Round2, all of the data is now freely available.

#### The AIMed corpus

The AIMed corpus [40] is now one of the most widely used corpora for protein-protein interaction extraction [27]. The original corpus consists of 230 PubMed abstracts. 200 abstracts are identified by the Database of Interacting Proteins (DIP) [41], as describing interactions between human proteins. The protein annotations are either parts of the protein interaction annotations, or are uninvolved in any protein interaction annotation. Since negative examples for protein interactions are rare in the mentioned 200 abstracts, the other 30 abstracts were manually selected. This would allow for the selected sentences to have more than one protein; however, the situation does not refer to any interaction (according to DIP). The currently released corpus consists of 225 abstracts (200 abstracts with positive examples and the other 25 abstracts with negative examples). In the release, there are 2,212 sentences and 4,084 protein references.

Due to the ambiguities involved in human gene/protein names, the creators of the AIMed corpus developed a set of conventions for consistent tagging [16].

#### The protein mention recognizer

The protein mention recognizer used in our work is a Maximum Entropy Markov Model n-best tagger [42]. To reduce our task to a simple linear sequential analysis problem, we performed some preprocessing on the corpora before using them in the experiments. For AIMed and GENIA, we removed all of the embedded tags, and only retained the outermost tags. There were 143 (3.4%) embedded occurrences in the AIMed corpus, five of which were triple-nested. There were 1,595 (1.7%) embedded cases in the GENIA corpus. For GENETAG, the alternatives were ignored, and the longest annotations were kept.

## Authors' contributions

JDK conceived the original idea. YW developed the idea, carried out all of the experiments, and wrote the manuscript. JDK, RS, SP and JT participated in discussion and revising the manuscript. All authors read and approved the final manuscript.

## Appendix - Sentences including the same annotated entities

The boldface represents an annotated entity, and in the GENIA examples, the word under the line represents the class used to annotate the entity.

### Protein_subunit

Thus, after 4 hr of exposure to  and , the expression of  was down-regulated,  was slightly up-regulated, while  remained largely unaffected. (GENIA PMID 7479924)

Interaction of **IL-2R beta **and gamma c chains with **Jak1 **and **Jak3**: implications for XSCID and XCID. (AIMed PMID 7973658)

Our results demonstrate that distinct cytoplasmic domains of these **cytokine receptors **elicit convergent signaling pathways and provide evidence that **beta c **and **IL-2R beta **function as a complete signal transducer. (GENETAG PMID 8721989)

### Protein_complex

 revealed the presence of a previously  for both the  and  at . (GENIA PMID 10022897)

The death domain of **tumor necrosis factor (TNF) receptor-1 **(**TNFR1**) triggers distinct signaling pathways leading to apoptosis and **NF-kappa B **activation through its interaction with the death domain protein **TRADD**. (AIMed PMID 8612133)

The decrease of **TNF receptors **by **IL-4 **was accompanied by down-regulation of TNF-induced activities, including cytotoxicity, **caspase-3 **activation, **NF-kappaB **and **AP-1 **activation, and **c-Jun N-terminal kinase **induction. (GENETAG PMID 9837907)

### Protein_domain_or_region

The  is an essential component of the receptors for  and . (GENIA PMID 9199305)

Interaction of **IL-2R beta **and gamma c chains with **Jak1 **and **Jak3**: implications for XSCID and XCID. (AIMed PMID 7973658)

Our results demonstrate that distinct cytoplasmic domains of these **cytokine receptors **elicit convergent signaling pathways and provide evidence that **beta c **and **IL-2R beta **function as a complete signal transducer. (GENETAG PMID 8721989)

## Supplementary Material

Additional file 1 **The boundary words of the GENIA, GENETAG and AIMed corpora**. The data is formatted as: annotation entropy|Part-Of-Speech|boundary word|the number of annotated occurrences|the number of un-annotated occurrences. Here, "NN Before" and "NN After" indicate boundary nouns that occur before and after protein names, respectively. Click here for file
